# Food insecurity in children and young people in Scotland

**DOI:** 10.1017/S0029665124000090

**Published:** 2024-01-22

**Authors:** Stephanie Chambers, Kathryn Machray, Gillian Fergie

**Affiliations:** 1 School of Social and Political Sciences, University of Glasgow, 28 Bute Gardens, Glasgow, G12 8RS; 2 MRC/CSO Social and Public Health Sciences Unit, School of Health and Wellbeing, University of Glasgow, Clarice Pears Building, 90 Byres Road, Glasgow, G12 8TB

**Keywords:** Food insecurity, child, adolescent, preschool, Scotland

## Abstract

The aim of this review was to highlight the key issues in relation to food insecurity among children and young people living in Scotland. It provides an overview of the current context of food insecurity more generally within the UK and specifically in Scotland. Food insecurity has risen in Scotland evidenced through responses to national surveys and the dramatic increase in households relying on emergency food provision. Food insecurity is highest among young people, single parent families and single men. The key drivers of food insecurity include insufficient income, welfare reform, food inflation and geo-political events. Evidence suggests that food insecurity is negatively related to sufficient nutritional intake, and the implications for physical and mental health are profound. Policy actions implemented to mitigate the impact of food insecurity on children and young people include the introduction of the Scottish Child Payment, food voucher schemes, free school meals, and holiday food provision. Further evidence is required to evaluate the success of these policies in reducing or mitigating food insecurity. The review concludes by considering the ways in which a rights-based approach to food might benefit children and young people living in Scotland, and argues that wider systemic change is required.

## Introduction

Food insecurity - the “limited access to food … due to lack of money or other resources”^1^ has risen dramatically across the UK, and particularly in Scotland^2–4^. Food insecurity can occur at the national level from external shocks or other structural issues impacting on the food system, but also within households when their ability to access nutritionally adequate and safe food is constrained or unavailable through uncertain or socially unaccepted means^5^. Food insecurity has been used interchangeably with food poverty. Food insecurity tends to focus on measuring the extent of the issue whilst food poverty describes the lived experiences of households on low incomes who may have limited access and ability to afford food^6^. Food poverty is not experienced alone: driven by income insecurity, it is experienced alongside (and in competition with) fuel poverty and other strains on household budgets. Food insecurity can be experienced as mild to moderate to severe^7^. Mild experiences might include anxiety around a household’s ability to obtain food. For moderate experiences households might compromise on diet quality or quantity. Severe food insecurity focuses on experiences of hunger. Hunger is the embodied sensation of severe food insecurity that goes beyond a temporary state.

Increased focus has been given to the nutritional status of children living Scotland. Childhood obesity rates have risen to 18%^8^ and there are growing inequalities between children living in Scotland’s most and least deprived areas^9^. In 2019/20 children living in the most deprived areas were twice as likely to be at risk of obesity than their peers living in the least deprived areas^9^. These inequalities are unlikely to be the result of social patterning in physical activity, where there are limited differences observed across deprivation levels^10^. It is likely therefore that the social patterning identified in nutritional intake^11^ is a major contributor to these inequalities in obesity outcomes. With health inequalities widening, it is vital that children and young people living in poverty are a key focus of population health concerns^9, 12^. These concerns have been recognised in Scottish policymaking. Scotland’s First Minister, Humza Yousaf, reiterated the Scottish Government’s commitment to reducing child poverty in a 2023 policy prospectus, highlighting policies aimed at increasing family incomes, supporting parents into employment and free school meals^13^. Addressing food insecurity is a measure through which the success of these policies could be judged.

The aim of this review is to highlight the key issues in relation to food insecurity among children and young people living in Scotland. It provides an overview of the current context of food insecurity more generally within the UK and specifically in Scotland. It considers who is impacted most, causes of insecurity, and the impacts of food insecurity on health and wellbeing. It then outlines some of the policy actions put in place to mitigate the impact of food insecurity, and the evidence for the effectiveness of these policies. The review concludes by considering the ways in which a human right to food might benefit children and young people living in Scotland.

## Context of food insecurity in the UK

### Increasing food insecurity

Those who are most socioeconomically disadvantaged are most likely to experience food insecurity. The Office for National Statistics reported that 61% of those living in the UK’s most deprived areas reported cutting down on food purchases compared with 44% of those living in the least deprived areas^2^. A poll from August 2022 found that 40% of people living in the UK in receipt of Universal Credit, a benefit for those on a low income or out of work, reported skipping meals over the previous three months^3^.

In September 2022, 26% of households in England with children reported experiencing food insecurity compared with 12.4% in May 2020^14^. Households reporting experiences of food insecurity are much more likely to report that they are concerned about the impact of being unable to afford sufficient food on their children’s mental and physical health, as well as the wider impact of the cost-of-living crisis on their children’s friendships and social development. Teaching staff have reported an increase in children arriving at school hungry and with lunches brought from home with insufficient food^15^. Teaching staff also support extending eligibility for Free School Meals^16^. Data on secondary school pupil registration for FSM in Scotland indicates that this has increased from 14.2% in 2016 to 18.6% in 2022 ^17^.

To manage experiences of food insecurity, those affected often rely on emergency food aid. The number of emergency food aid parcels distributed has increased substantially in recent years. For example, the Trussell Trust network (the largest emergency food aid network in the UK) reports that the number of parcels they distributed from April-September 2022 rose by almost one third ^18^. Similar percentage increases are reported for the numbers of parcels distributed for children (rising from 368,160 April-September 2021 to 483,677 April-September 2022). In Scotland during the same time period, 116,000 emergency food parcels were distributed, with 40,000 parcels for children ^4^. Households with children are overrepresented in referrals to Trussell Trust food banks ^19^, despite the Scottish Government’s introduction of a Scottish Child Payment. From February 2021, this provided low-income households with children under 6 years with an additional (at that time) £20 per week for each child. From November 2022 this increased to £25 for each child under 16 years for eligible families.

## Who is impacted most?

Single parent families (who are more likely to be headed by women) are the group most likely to report experiencing food insecurity^8^. For single parents, 34% reported that lack of resources had left them worried that they might run out of food during the previous 12 months, with 12% reporting that they had run out of food. Single parents (predominantly single mothers) and single men are also the most frequent users of food banks^20–23^. There is a lack of data available on levels of food insecurity among CYP, in part due to the household level focus of data collection in this area. A Food Foundation survey has highlighted the differences in levels of food insecurity between households with (23.4%) and without children (14.8%)^24^.

## Causes of food insecurity

### Insufficient income

A range of causes have been identified to explain increases in food insecurity across Scotland and the UK. The main overarching explanation is insufficient income^19, 25^. This can arise from both wages and benefits being too low, particularly in the context of rising costs. In-work poverty has increased in the last 25 years. In 1996/7, 44% of children and working-age adults were living in families where at least one adult was working. By 2019/20 this had increased to 66%^26^. A main driver of insufficient income is a labour market in which wages have not risen in line with inflation^27, 28^. This limits families’ spending power, making some of the most basic necessities, such as food and fuel, a challenge to purchase in sufficient quantities.

Families on low-incomes are at increased risk of experiencing food insecurity. After housing and energy, food spending is likely to take up the greatest proportion of these household’s outgoings^29, 30^. Evidence indicates that amongst food bank users in the UK, low to no income is common^21, 31^. It is estimated that 94% of people who use a food bank are living in destitution^23^. Low-income households often consider food spending to be a flexible expense and will prioritise other spending ahead of food expenditure, such as housing^32^. The impact of this is that families on low incomes need to spend a higher proportion of their income in order to eat a diet in line with dietary recommendations^33^.

### Welfare reform

Families in receipt of benefits are also impacted by having insufficient income to meet their needs. Following the global financial crash in 2008, the Conservative-Liberal Democrat coalition government introduced a range of austerity policies, cutting public spending and freezing benefits. Additional changes were introduced through the Welfare Reform Act which reformed benefits in the following years including capping benefits for personal independence payments, limiting housing benefit payments when households had extra bedrooms, payments for child benefit being limited to only two children per household, increased conditionality and sanctions, and the move towards the Universal Credit system. Universal Credit has been identified as a key explanation for increases in food insecurity, particularly the introduction of a five-week wait for claimants to receive their first payments. This has increased the likelihood that claimants are indebted to the Department of Work and Pensions (DWP), putting further pressure on household budgets. Bennett-Clemmow *et al*.^34^ identified that 47% of people referred to a Trussell Trust food bank were indebted to the DWP. These policies and changes to the welfare system have been identified as key causes for increases in food insecurity^21, 35–38^. For example, Loopstra *et al*.^39^ highlighted that as benefits reduce, higher rates of food parcel distribution are seen across local authorities in Scotland, Wales and England. In a later study, Loopstra *et al*.^40^, found that sanctioning of benefit claimants was positively associated with increased food bank use. Despite significant media attention on such reforms, and various experiences of poverty^41^, these issues remain.

### Food prices

From mid-2021 food prices started to increase steeply in the UK, with inflation levels peaking at 19.2% in March 2023^42^. Of particular concern has been the above average increase for staple foods such as milk, cheese, eggs, pasta and oil^2^. As would be expected, the increase in the cost of food is associated with an increase in the number of households reporting that they have experienced food insecurity. In August 2020, 6.9% of UK households reported moderate or severe food insecurity compared with 18.4% of households in September 2022^24^. In 2021, 9% of adults responding to the Scottish Health Survey reported that they had been worried that they would run out of food because of a lack of money or other resources in the last 12 months^8^. Food insecurity figures for Scotland have not yet been updated to take into account the highest levels of food inflation.

### Geo-political events

Additional challenges are geo-political events. In January 2020, the UK left the European Union (EU), leading to disruptions in food supply chains^43^. Issues have included temporary food shortages and higher food prices. Food and energy prices have also come under considerable pressure after Russia invaded Ukraine in February 2022. With export of goods restricted from Ukraine and Russia, prices of staples such as wheat and oils have increased, as have the food products that include these ingredients^2^. The invasion has also impacted fuel supplies, coinciding with the Energy Price Cap increasing, and average household electricity and gas payments in the UK have almost doubled^44^, making cooking even more challenging.

## Food insecurity and diet quality

Being food insecure increases the likelihood that individuals will consume diets that are of a lower quality^45^. Food insecurity is associated with below average intakes of fruits, vegetables and fish^46^, and a less diverse diet^47^. Children living in food insecure households are at higher risk of having diets of poorer nutritional quality, with lower consumption of vegetables and higher consumption of nutrient poor highly processed foods^48–50^. Micronutrient intakes have been identified as insufficient in food insecure children, with vitamin D, magnesium and calcium intakes lower than intakes among food secure children^51^.

Emergency food provision generally does not provide diets of sufficient nutritional quality^45^. Food provisions available from food banks often do not provide food with sufficient energy, are high in sugar, and the products provided lacking in fruit and vegetables, iron, calcium, zinc and vitamins A, D, C and E^45, 52, 53^. Where meals are provided, they also tend to be high in fat, sugar and salt and low in key nutrients, such as vitamin D^53, 54^. For organisations involved in distributing emergency food, their main concerns can be to address the immediate crisis situation for households facing food insecurity and reduce the more immediate psychological damage rather than mitigating longer term nutritional risks^52^.

## Impact of food insecurity on health

Inevitably if those experiencing food insecurity are not eating a diet in line with recommendations, there is a greater likelihood that their health will be negatively impacted. Food insecurity is associated with conditions including, diabetes, obesity, metabolic syndrome and inflammation ^40^. Bramley *et al*. ^23^ found amongst a UK sample that those reporting poor health were six times more likely be food insecure than those reporting excellent health. Evidence suggests that children experiencing food insecurity are more likely to report poorer health, more hospitalisations, under or overweight, developmental delays, and to be at greater risk of anaemia and asthma than children who are food secure^55–58^.

A particular challenge is when those experiencing food insecurity rely on diet to manage chronic health conditions^59, 60^. These conditions can include higher blood pressures, type 2 diabetes and heart disease^60–64^. In children, type 1 diabetes is a chronic health condition that food insecurity makes harder to manage^65–67^. The relationship between food insecurity and health is bi-directional. Poor health also leads to food insecurity with those unable to work often living on insufficient incomes, as well as the additional costs incurred for people who are unwell or disabled^68^.

Food insecurity is also associated with poor mental health, including depression, anxiety, stress, suicidal ideation and mood disorders^69–74^. Severe food insecurity is associated with poorer mental health outcomes^72^. Cohort studies in the UK have found that food insecurity increases risk of poor mental health in pregnant women^75^ and in low-income families with children ^76^, highlighting families’ vulnerabilities. The lived experience of those reporting food insecurity and poor mental health has been explored qualitatively too^59, 77, 78^. Participants in these studies have discussed the difficulties of uncertainty, worry, stress and lack of food choice and access to appetising food as all impacting on their mental health. In children, an association has been identified between food insecurity and social and emotional wellbeing, with higher risk of behavioural problems, negative health perceptions, life dissatisfaction, anxiety and depression for the children affected^79–81^. Poor health in childhood is associated with poor health in adulthood^82^, highlighting the long term impact of childhood food insecurity.

## Impact of food insecurity on other outcomes

For CYP, the physical health impacts of food insecurity may not arise until adulthood. Nonetheless other impacts are experienced in the shorter term. Across children’s age ranges, food insecurity has a negative impact on behavioural, academic and emotional outcomes^83, 84^. For young people in post-secondary education there is evidence that food insecurity is associated with lower academic performance and lower retention^83^. In qualitative explorations with parents, they described being unable to provide the diet they would choose for their children^85, 86^. Parents reflected that the diet they were providing for their children was lower quality in comparison to their own diet at the same age, with repetitive meals and low value products of poor nutritional quality^85^.

Research with children and young people around food insecurity in the UK is relatively limited. In Harvey’s study^85^, she spoke with children as well as parents. Children said their parents were not always able to shield them from the impact of food insecurity, with weekends without school food particularly challenging for some. The social impact of food insecurity has been identified as a real concern for children and young people. Sharing of food is an opportunity to build social bonds^86–88^. Children are acutely aware of when their experiences are different from others in their peer groups, and the portrayal of eating a diet as ‘normal’ as possible becomes a key strategy that they pursue^89^. Evidence from beyond the UK has shown that when this is not possible, food insecurity impacts on children and young people’s emotional wellbeing^90–93^.

## Policy actions to reduce food insecurity in children and young people

Few specific policy actions have been put in place to address food insecurity in the adult population. The UK Government’s decision to increase benefits by the rate of inflation in 2023 is likely to have mitigated the levels of food insecurity that were likely to be seen had these benefits increased at the same rate as wages^24^. Levels of food insecurity of households in receipt of Universal Credit have stabilised in 2023 at 48-49% compared with a high of almost 54% in 2022^94^. The Scottish Welfare Fund is a source of emergency funding that households can apply to that provides them with additional money within a relatively short space of time. There are concerns, however, that those on a low-income are having to rely on this fund on an ongoing, rather than emergency, basis^95^.

### Target populations for policy

Schneider and Ingram^96^ created a conceptual scheme to group target populations for policies. Within this scheme, each target population can be placed in one of four categories based on whether that group is constructed either positively or negatively in relation to a policy problem, and as either weak or strong in relation to the power they hold in the policy arena. This results in four different types of target populations: 1) advantaged (positive and strong – elderly, business, scientists); 2) contenders (negative and strong – rich people, unions, cultural elite); 3) Dependents (positive and weak – children, mothers, disabled people); 4) Deviants (negative and weak – criminals, drug addicts). They argue that support for policies is likely to be higher among both the public and policymakers where the target population is constructed positively. In terms of food insecurity, the scheme generates hypotheses for why a greater number of policies appear targeted at children and families compared with others who are experiencing food insecurity, such as single men. Indeed, the majority of adults in the UK believe that Governments have a responsibility to support children, and support the extension of policies such as free school meals to achieve this^97–99^.

### Scottish Child Payment

In 2021, the Scottish Government introduced the *Scottish Child Payment* with the long-term aim of reducing child poverty. This was initially a payment of £10 per week for families receiving certain means-tested benefits for each child they were caring for under 6 years of age. The payment was introduced in November 2020 and was doubled to £20 in April 2021, during the Covid-19 pandemic. The payment has been increased to £25 per week per child under 16 years of age, and is in addition to Child Benefit Payments, which are also available to families living elsewhere in the UK. Families are paid 4-weekly and are able to spend the money in the ways they deem best. It is estimated that take-up of the benefit is only around 77%^100^. In a qualitative study, Scottish Child Payment recipients (n=39) from across Scotland reported using the money to buy food helped them avoid falling into debt, or prevented them from having rely on emergency food^100^.

### Food assistance

In England, Wales and Northern Ireland, these targeted policies include *Healthy Start* vouchers for women who are 10 weeks or more pregnant and families who have children under the age of four. These vouchers allow families to purchase vitamins, milk, fresh, frozen, and tinned fruit and vegetables, fresh, dried, and tinned pulses, and infant formula milk. Scotland runs a similar scheme called *Best Start Foods* where families are provided with pre-payment cards to purchase healthy foods including milk, infant formula milk, vegetables, pulses and eggs. Evaluation of these schemes is currently limited, but they have been identified as key areas for future evaluation^101^. The limited quantitative evaluations that have been carried out have not focused on food insecurity. The outcomes that evaluations have focused on have shown mixed results. For example, Parnham *et al*.^102^ analysed pooled Living Costs and Food Survey (LCFS) data from 2010-2017 and concluded that *Healthy Start* had not increased fruit and vegetable expenditure for participating families compared with non-participating eligible families. In contrast, using a longitudinal design with 296 households, Griffith *et al*.^103^ found that fruit and vegetable expenditure increased between December 2004-November 2006 to December-November 2008, and that the nutritional quality of food purchases improved. Families describing the impact of *Healthy Start* have identified the scheme as helping to alleviate hunger^104^ and to help women buy healthy food during pregnancy^105^. Multiple explanations can be attributed to these differences including study design (pooled cross-sectional vs longitudinal) as well as the differing time periods and data sources (LCFS vs Kantar panel data). At this time, there have been no evaluations of *Best Start Foods*.

Beyond the UK, food assistance programmes, such as the Special Supplemental Nutrition Program for Women Infants and Children (WIC) and the Supplemental Nutrition Assistance Program (SNAP) in the USA, have been shown to prevent or mitigate household and child food insecurity, reduce the risk of caregiver and child poor health^106, 107^. Evidence suggests that the health and economic benefits of SNAP are long lasting with children continuing to benefit into adulthood^108^.

### School meals

The other main policies which aim to mitigate food insecurity are free school meals (FSM). School meals have a long history in the UK, beginning in the 19^th^ century with charitable organisations providing meals to children whose families were unable to feed them. Manchester was the first municipality to provide FSM to children requiring them. Following concerns around nutritional intakes and the physical condition of conscripts to the Boer War, the 1906 Education Act allowed for local authorities to provide free school meals to children during the school day, though very few did^109^. Following World War 2, and the expansion of the welfare state, these duties became statutory and nutritional standards for school meals were set^110^. In the 1980s, compulsive competitive tendering and the abolition of nutritional standards saw a decline in the nutritional quality of school meals^110^. Eligibility criteria also changed during this time resulting in over 500,000 children from low-income households becoming ineligible to receive a free school meal^111^.

With a focus on rising levels of obesity, and concerns around children’s nutritional intake, food and nutrient standards have been re-introduced across the devolved nations of the UK. In the early 2000s, the Scottish Government published the Hungry for Success^112^ guidance document, followed soon after by school food regulations in 2007^113^. These were subsequently updated in 2021 to further reduce levels of sugar, improve the nutritional quality of drinks, and reduce the frequency of red and processed meat^114^. In England, school food standards are in place and have been mandatory since 2008 for primary schools and in 2009 for middle and secondary schools, and updated standards were introduced in 2015^115, 116^. The introduction of these standards and regulations have been shown to have improved the nutritional quality of school meals in England^117–120^, but evidence is limited from Scotland.

Until 2014, FSMs were provided on a means-tested basis across the UK for families in receipt of certain benefits or who had a low annual income. With the Liberal Democrat-Conservative government elected in 2010, a universal FSM system for children in their first three years of primary school was introduced in 2014 in England and in 2015 in Scotland. This had been a Liberal Democrat manifesto commitment in 2010. Wales rolled out a universal free breakfast programme to all primary-school aged children, whilst Northern Ireland retained the means-tested system, although with wider eligibility criteria. Some of the key policy aims of universal free school meals (UFSM) was to reduce the stigma associated with the means-tested system and to improve children’s health and wellbeing through the provision of healthy food. Interestingly, mitigating food insecurity was less likely to be discussed as a policy aim, though it was recognised as a likely outcome by some key stakeholders involved in delivering the policy^121^. As outlined above, since the introduction of the policy, reported food insecurity and use of emergency food provision has increased massively. This has led for calls for UFSM to be introduced more widely to either all primary school children or all school children, including those in secondary schools. It has been estimated that 800,000 children living in poverty are not eligible to receive FSM under a means-tested system, and almost a quarter of children not receiving a FSM during Covid-19 restrictions were food insecure^99, 122^. The Scottish Government pledged to widen universal provision to all primary school children within the 2021-2025 Scottish Parliament. By 2023, this had only been extended to children in the first five years of primary school, with the capital infrastructure needed for full implementation identified as a barrier by Scotland’s First Minister^123^. Universal provision was implemented for children receiving free childcare places in early years settings in Scotland by 2021. The Welsh Government announced in 2023 that universal free school lunches would be available to all primary school pupils, having previously only had a universal service for breakfast. In February 2023, the Mayor of London announced that all primary school children in the capital would be eligible to receive a FSM for the 2023/24, with five boroughs already providing this offering. The main aim outlined for the policy was to provide emergency support to families during the cost-of-living crisis, and to reduce families’ food insecurity^124^. There have been calls from third sector organisations working in the areas of poverty alleviation, family support and food to extend FSM eligibility to all children whose families are in receipt of Universal Credit and/or to move to universal provision at primary and secondary level^98, 99^.

Ahead of universal provision being introduced for the youngest primary school-aged children, pilots were carried out in Scotland and England. Evaluations of these pilots reported the perceived financial benefit of FSM for families and increases in attainment in England^125–127^. They also indicated that universal FSM had reduced barriers, including stigma, and increased uptake for children who were eligible to receive FSM under the means-tested system^128^. The impact on food insecurity was not addressed in the pilots, or directly in the evaluations of the policies’ rollouts in both countries. Evaluations of the fully implemented policy in Scotland show that there has been no impact on attendance or behaviour^129^, however, parents perceived a financial benefit^130^. Positive outcomes observed in England include increased take-up in previously eligible FSM children, reduction in absence inequalities between children from higher and lower income backgrounds, reductions in obesity, financial benefits for families, and perceived improved readiness for learning^131, 132^. Evidence suggests that the policies in both England and Scotland have lowered the consumption of crisps, total fat and sodium with the biggest impacts seen in children from families on low incomes^133^. These studies focus on relatively short-term outcomes given the recent implementation of these policies, however, a forecasting study examining the cost-benefits of universal provision for all children were such that for every £1 invested between 2025-2045, a return of £1.71 could be expected, with the potential for wider net benefits of £58 billion to the wider economy^134^. There is some evidence from Wales that children at higher risk from food insecurity are more likely to take-up a breakfast offering and are less likely to skip breakfast under the universal scheme^135^. The findings of the evaluations of UFSM suggest that families facing food insecurity are likely to be benefitting from its implementation, however, it is challenging in the short term to assess whether they have reduced or mitigated food insecurity within the context of wider societal issues.

Beyond the UK, and as other areas, particularly within the United States, have introduced universal or have extended existing provision, more evidence of the impact on outcomes has been produced. These benefits include increased fruit and vegetable consumption, improvements in diet quality, improvements in educational difficulties and academic performance, financial benefits for families, reduced obesity risk, reduced health inequalities and reduced food insecurity risk^136–140^. Holley & Mason^137^ have flagged some of the limitations of these studies, including sample sizes being relatively small in experimental studies, along with incomplete outcome measures, lack of consistency across cohort studies in terms of addressing representativeness, and limited input from children in qualitative evaluations. An additional limitation is that measured outcomes are, as yet, relatively short term, and more time is needed to fully measure the impact of UFSM, particularly on improving health in childhood and beyond.

FSM provision has focused on children in early years or primary school rather than young people attending secondary education. Organisations such as the Scottish Trades Union Congress ^141^ have campaigned for the Scottish Government to extend universal provision to secondary school pupils in light of the cost-of-living crisis and rising food insecurity. A study carried out by Chambers *et al*.^110^ found that where more young people were eating a school meal, take up amongst those registered for a FSM was higher. In addition, where the proportion of young people registered for a FSM were higher, take up was also higher among those young people. Interestingly this study indicated that levels of school modernisation did not seem to be associated with take-up, highlighting the importance of social connections for older children in relation to food consumption in schools. Pilots of UFSM at secondary level have run in some limited areas. In Hammersmith and Fulham, a London borough, two secondary schools offered universal FSM as a pilot in 2019. Key findings from evaluation are that uptake increased in young people previously eligible under the means-tested system, teachers reported improved behaviour and increased readiness to learn in the afternoon, and stakeholders perceived that food insecurity had been reduced^142^. The FUEL study, currently underway in England, led by the University of Birmingham and funded by the National Institute for Health Research (NIHR), is examining whether School Food Standards improve secondary school pupils’ dietary intake^143^. Work more specifically aimed at answering questions around food insecurity is underway through the CANTEEN study also funded by NIHR and led by Queen’s University Belfast. The study is examining the role of FSM in addressing food insecurity and diet quality in secondary school pupils (https://fundingawards.nihr.ac.uk/award/NIHR151295). The mixed methods study aims to evaluate the effectiveness and cost-effectiveness of FSM in UK secondary schools on nutritional quality and food insecurity. It will evaluate the association between school-level FSM uptake/individual level uptake on these key outcomes, with economic and process evaluations also undertaken. The findings of these studies will contribute to an up-to-date understanding of the effectiveness of FSM policy in addressing food insecurity and dietary quality.

### Out of school food

With the introduction of austerity measures following the global financial crash, concerns were raised about the risk of food insecurity among children during school holidays. This was described as ‘Holiday Hunger’. An All-Party Parliamentary Inquiry into Hunger was chaired by Labour MP Frank Field, whose report highlighted that school holidays were putting 3 million children at risk of hunger^27^. The issue was put under the spotlight even more by the school closures resulting from the Covid-19 pandemic. Provision of free school meals during this time came under substantial scrutiny as families shared photographs of woefully inadequate deliveries of food for children to eat over the week for lunch. Manchester United footballer Marcus Rashford galvanised a campaign to address inadequate food provision during this time, putting pressure on government to not only improve provision, but also to commit to providing food over the school holidays to families most in need^144^.

When the UK Government rolled out a voucher scheme for children in England in receipt of FSM, many families were unable to access the electronic system^94^ or struggled with digital literacy^145^. It is estimated that almost half of eligible children did not receive any form of FSM during the Covid-19 lockdown^146^. The UK Government announced that the voucher scheme would stop during the school summer holidays of 2020. A legal challenge was mounted by the Non-Governmental Organisation, Sustain, and the Good Law Project to challenge this decision. The UK Government reneged and therefore never took the opportunity to clarify Government responsibility for food provision in court^146^.

The policy response to these concerns, and others around nutritional intake, physical inactivity, and social isolation, has been to introduce means through which families can access food, either through vouchers/direct payments or through holiday programmes that provide food. In England, the Holiday Activities and Food Programme rolled out from 2018 to 2025. Although annual evaluations of the programme have been carried out, the impact on food insecurity has not been measured, as this is considered a possible additional impact, and is not one of the main outcomes identified in the programme’s logic model^147^. Instead, the focus has been on nutrition, healthy behaviours and educational outcomes. This highlights that food insecurity among CYP’s has tended to be a secondary focus of programmes that might mitigate its impact.

Evaluations of the impact of food provision during holidays on food insecurity have been limited, but there is some evidence to suggest programmes can make a difference. These studies suggest positive impacts, but they have been small in scale. A pilot study^25^ surveying parents found that 42% of children participating in a holiday club in the UK were from insecure households, with 24% reporting food insecurity with hunger. The results indicated that food insecure households reported benefiting most from the club in terms of it improving their ability to access food during school holidays. Holley & Mason ^148^ report similar findings from research with both holiday programme staff and attendees. In a qualitative study, participants identified that the programme had provided not only social and financial benefits, but also had improved nutrition and attenuated hunger. Much more work is required to understand the extent to which holiday programmes can address food insecurity, or whether programmes that provide families with transfers of money are more effective.

## Right to food

The concerns outlined in this review around food security have led to calls for the right to food to be enshrined in UK legislation. Whilst it is enshrined in international laws to which the UK is a signatory, international law is harder to enforce than domestic law^149^. A right to food includes having adequate, safe, accessible, nutritious food, to be free from hunger, and that methods of food production, conservation and distribution are improved^149^. These calls are not new: more than 20 years ago, Dowler & Caraher^150^ called for food access and consumption to be framed as core tenants of citizenship. Although food plays a defining role in promoting and maintaining our physical health, it is also a means through which we participate in society and build social relationships. When individuals or families are unable to do this through food insecurity, their social exclusion increases^151, 152^. Within international treaties, the link between food and the dignity of the human person is outlined^149^. Food is framed as being more than a nutritional need, and as inherently important to realising other human rights.

The UK Government has been accused of failing in its duty to realise the right to adequate food^153^, evidenced through the rise in the number of food banks^4, 18^. This has been identified particularly for children, where the Special Rapporteur on extreme poverty and human rights and the Special Rapporteur on the right to food wrote joining to the UK government expressing concern for:

the deepening level of food insecurity among low-income households, particularly families with children, and the lack of comprehensive measures to ensure their access to adequate food ^154^.

The Good Food Nation Act sets out the official responsibilities of the Scottish Government, local authorities and other public bodies to ensure that adequate access to food is available to all^155^. The right to food has not yet been enshrined in law, however. It is intended that the right to food in Scots law will be enshrined within the forthcoming Human Rights Bill. The Scottish Government has argued that this means that the right to food is approached within the framework of other human rights. Potential challenges to this are that this intersects with areas of policy reserved to the UK Government, such as social security^149^. Another route through which children’s right to food could be enshrined in Scots law is through the United Nations Convention on the Rights of the Child (UNCRC) (Incorporation) Bill. The progression of the Bill through parliament has currently been paused by the UK Supreme Court through concern that it may allow Scottish Courts to override Acts of Parliament that contravene the UNCRC. It is likely however that the Bill will be revised and incorporated into law, providing greater protection for children in Scotland to food that is available, accessible and adequate^156^. How this right will be implemented remains to seen, but O’Connell & Brannen^157^ argue that a well regulated publicly funded school meal system that offers children the equal opportunity to eat nutritious food, without fear of exclusion, is a means through which children’s right to food can be realised. Whilst school meals can be a means through which food insecurity may be mitigated in the school setting, it can only ever be partial given the range of other settings, institutions and social practices that impact on CYP’s access to and experiences of food.

## Conclusion

This review has outlined the challenges families in Scotland and the rest of the UK are facing in feeding their children. Food inflation has risen steeply within the context of a wider cost-of-living crisis. Single parent families are at particularly high risk of experiencing food insecurity. Policy action around food insecurity has been more focused on families with children than other population groups. Many of these policies offer food directly or indirectly, rather than addressing the main drivers of food inequality, such as inadequate incomes and rising costs. Policies like the *Scottish Child Payment* are exceptions, but without wider systemic change their impacts might be limited if levels of absolute and relative poverty are maintained. There is insufficient evidence at this time to assess whether these policies have been successful at reducing or mitigating food insecurity. The long-term impact of food insecurity needs to be closely monitored, as well as its contribution to health inequalities. This review concluded focusing on the adoption of a right to food within Scotland. Whilst there have been moves towards this, it has not yet been realised. Enshrining the right to food in legislation would clarify governments’ responsibilities in ensuring that all citizens, including children, are able to access adequate food. This needs implementation alongside wider system change to ensure that families also have adequate incomes to lift them out of all forms of poverty, not only food.
